# Immigrant enclaves and risk of diabetes: a prospective study

**DOI:** 10.1186/1471-2458-14-1093

**Published:** 2014-10-22

**Authors:** Briana Mezuk, Klas Cederin, Xinjun Li, Kristen Rice, Kenneth S Kendler, Jan Sundquist, Kristina Sundquist

**Affiliations:** Department of Family Medicine and Population Health, Division of Epidemiology, Virginia Commonwealth University School of Medicine, P.O. Box 980212, Richmond, VA 23298 USA; Virginia Institute for Psychiatric and Behavioral Genetics, Virginia Commonwealth University School of Medicine, P.O. Box 980126, Richmond, VA 23298 USA; Centre for Primary Health Care Research, Lund University, Malmö, Sweden

## Abstract

**Background:**

The diversity of the Swedish population has increased substantially over the past three decades. The aim of this study was to assess whether living in an ethnic enclave is associated with risk of diabetes mellitus (DM) among first and second-generation immigrants and native Swedes.

**Methods:**

Cumulative incidence of DM in three urban municipalities was assessed from 2006–2010 by linking records from the national census, multi-generational family register, and prescription drug register. Immigrant enclaves were identified using Moran’s Index. Multi-level logistic regression was used to assess the relationship between enclave residence and risk of DM for three groups: Iraqi immigrants, non-Iraqi immigrants, and native Swedes (N = 887,603).

**Results:**

The cumulative incidence of DM was greater in Iraqi enclaves compared to other neighborhoods (4.7% vs. 2.3%). Among Iraqi immigrants, enclave residence was not associated with odds of DM (Odds ratio (OR): 1.03, 95% Confidence Interval (CI): 0.86 – 1.24). Among other immigrants, enclave residence was not associated with DM after accounting for neighborhood deprivation. Among native Swedes, enclave residence was associated with elevated risk of DM even after accounting for neighborhood deprivation and individual-level characteristics (OR: 1.23, 95% CI: 1.11 – 1.36).

**Conclusions:**

Residential ethnic composition is associated with DM but this relationship differs across ethnic group. Enclave residence is not associated with increased odds of DM for immigrants, regardless of their nation of origin, but it is associated with increased likelihood of DM for native Swedes.

## Background

In 2000, over 171 million adults had diabetes mellitus (DM) worldwide, and the prevalence of this condition is projected to increase substantially over the next 15 years [1]. DM is a leading cause of morbidity and mortality, and is projected to be among the top 10 causes of mortality worldwide by 2030 [2]. Identifying modifiable risk factors for DM, particularly type 2 DM, which accounts for >95% of cases and whose incidence increases substantially in mid- and late-life [3], is a pressing public health concern [4].

There is a growing appreciation of the role that contextual environmental factors (e.g., “neighborhood” factors) may have on health. Neighborhoods have both physical (e.g., access to services, greenspace, availability of grocery, alcohol and tobacco outlets) and social (e.g., community unemployment, segregation, social capital, crime) attributes that may influence health [5]. Contextual environmental factors may influence health by placing constraints on (or promoting) health-related behaviors (e.g., smoking, alcohol use, poor diet, physical inactivity), or through acting as a source of (or buffer against) stressors [5]. A handful of studies have prospectively examined contextual environmental characteristics and risk of DM, with mixed results. Neighborhood deprivation and attributes of the physical environment (e.g. resources for physical activity, access to healthy food) have been associated with type 2 DM [6, 7] and related conditions, including obesity [8, 9], insulin resistance [10], and clinical indicators of persons with type 2 DM [11]. Other contextual factors, such as neighborhood ethnic composition – the degree to which one’s neighbors are of the same ethnic background as oneself – have also emerged as potentially important factors for health [12].

Ethnic enclaves may be particularly important to the health of immigrants because they provide a resource in terms of cultural goods, language, and kinship or social networks [13]. Enclaves are generally conceptualized as transitional spaces, in which individuals or their offspring leave as they become more acculturated to the host nation [14]; in this sense the relationship between living in an enclave and socioeconomic and health outcomes is expected to change over time and across generations [15]. For example, immigrant enclaves have been associated with better economic outcomes short-term, particularly for low-skilled workers [16]. However spatial assimilation is generally associated with long-term economic stability [17]. Alternatively, Logan et al. [13] defined an ethnic community as one in which ethnic minorities, particularly second generation and beyond, choose to live in despite having the ability to live in a more integrated setting. While immigrants living in an ethnic enclave may be doing so out of economic necessity, those continuing to reside in these settings may be doing so out of social preference and as a means of resisting cultural assimilation [18, 19]. This process of spatial assimilation varies substantially across different immigration groups [19, 20] and has changed over time as the source of immigrants to the US and Western Europe has shifted from Eastern European countries to Asian, African, and Latin American nations [20].

The relationship between residential ethnic composition and risk factors for DM is complex and appears to vary by ethnic group. In a study of Hispanic and Chinese adults in four US urban areas Osypuk and colleagues [12] found that the proportion of immigrants in a neighborhood was inversely related to consumption of high-fat foods but also sedentary behavior. Kershaw et al. [21] found that living in a neighborhood with a high concentration of people of one’s own race/ethnicity was positively associated with obesity among US black women, but inversely related to obesity for Mexican-American women. In New York City, living in a predominantly black neighborhood was inversely associated with hypertension among older foreign-born blacks, but was unrelated to hypertension status among US-born and younger foreign-born blacks [22]. In an Australian study, Astell-Burt et al. [23] reported that residing in an ethnic enclave was associated with lower body mass index for some, but not all, immigrant groups. Nobari et al. [24] argued that residing in an immigrant enclave may influence diet and physical activity behaviors via social networks, support, and norms.

The diversity of the Swedish population has increased substantially in the past 30 years. Today nearly one in five Swedish residents is of foreign nationality [25]. Sweden has become one of the main accepters of asylum seekers, most recently from conflict areas of the Middle East such as Iraq [25]. Parallel to this influx of immigration, income inequality has grown faster in Sweden than in any other industrialized country in the past 25 years [26]. The aim of this study was to assess whether living in an ethnic enclave is associated with risk of DM among first and second-generation immigrants and native Swedes. This analysis focuses on Iraqi immigrants because this population is the largest non-European migrant group in Sweden during our study period, and the largest among asylum seekers [27]. Also, Iraqi immigrants are among the least integrated migrant groups in the country [28]. We chose to focus on a single nationality in order to account for migration history differences that vary across immigrant groups. In sum, the confluence of increasing diversity and income inequality makes Sweden a particularly relevant place to investigate the relationship between ethnic enclaves and health.

## Methods

### Sample

The sample was based on nationwide registry data located at the Center for Primary Health Care Research at Lund University in Malmö, Sweden [29–31]. National census data in 2005 (e.g., age, sex, marital status, education, household income and country of birth) was linked to a Geographic Information Systems (GIS) database which covers the entire nation. All residential addresses in Sweden have been geocoded to small geographic units that have boundaries defined by homogeneous types of buildings. These neighborhood areas, called Small Area Market Statistics (SAMS), have an average of 1000 people (2000 in the Stockholm area) and were used as the primary unit to approximate neighborhoods [32]. These data were then linked to the Swedish Presciprtion Drug Register, which reports all medication perscribed and dispensed nation-wide [33]. All linkages were performed by the use of an individual national identification number that is assigned to each person (including immigrants) in Sweden for their lifetime, which was replaced by a random serial number for analysis in order to provide anonymity.

Swedish census records indicate country of birth but do not assess other characteristics of race/ethnicity, unlike US census data. In order to identify the three subpopulations of interest – 1st and 2nd generation Iraqi immigrants, 1st and 2nd generation immigrants from other nations, and native Swedes – the Census data was linked to the Multi-Generational Registry. The Multi-Generational Registry identifies each individual’s mother and father (if known) and country of birth. ‘Native Swedes’ were defined as individuals who were born in Sweden (2nd generation) and both of whose parents were also born in Sweden (1st generation). Iraqi immigrants were defined as individuals who were either born in Iraq (1st generation) or who had at least one parent who was born in Iraq (2nd generation). Similarly, members of other immigrant groups were defined as individuals who were either born outside of Sweden (1st generation) or who had at least one parent who was born outside of Sweden (2nd generation). The 10 most common non-Iraqi immigrant groups represented in this analyses migrated to Sweden from Finland (25%), countries in Asia (other than Turkey, Iran or Iraq) (10.9%), countries in Africa (8.5%), Yugoslavia (7.2%), Iran (6.4%), Poland (5.0%), Turkey (4.0%), Bosnia (3.8%), Chile (2.7%) and countries in South America other than Chile (2.7%).

This analysis is restricted to the three most popula-ted municipalities in Sweden: Stockholm, Malmö, and Gothenburg; approximately 17.9% of the Swedish population resides in these areas. The total number of SAMS included in the present study was 1490. The sample is restricted to individuals aged 30 and older by January 1, 2006, with no history of DM (N = 887,603).

This study is approved by the Institutional Review Board at Lund University.

### Measures

#### Immigrant enclaves

Iraqi immigrant enclaves were defined using GIS methods. The number of the Iraqi immigrants (1st or 2nd generation) in each SAMS was used to identify ethnic enclaves using Moran’s Index [34, 35]. Moran’s Index is a measure of relative similarity across an area (spatial autocorrelation), simultaneously taking its characteristics and location in consideration, that ranges from -1 to +1 (range for the present study: -0.03 to +0.38). Positive values of the Index mean that a neighborhood characteristic (e.g., number of Iraqi immigrants living in a given SAMS) is more similar to those of its neighboring areas than expected under the null hypothesis of a random distribution of attributes. This SAMS is then considered part of a cluster, or enclave. Negative values of the Index indicate that a SAMS is more dissimilar to those of its neighboring areas under the null hypothesis; this SAMS would be identified as an outlier. The values on the Index are then transformed into z-scores with an associated *P*-value. Only positive clusters with a *P*-value <0.05 were considered as indicative of an enclave.

**Figure 1:**
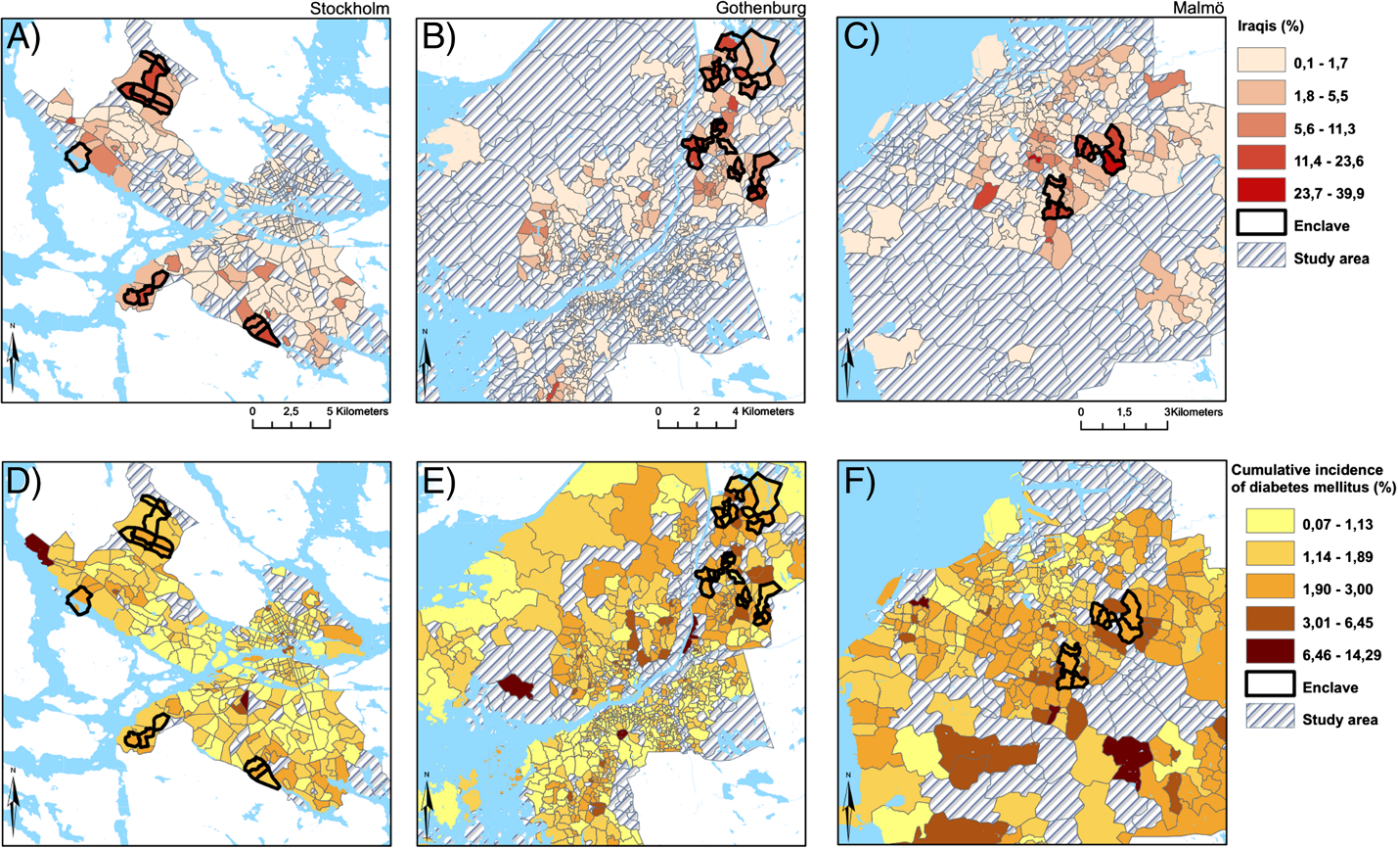
**Iraqi enclaves and Type 2 Diabetes Mellitus incidence.** Iraqi enclaves **(panels A-C)** and Type 2 Diabetes Mellitus incidence **(panels D-F)** in Stockholm, Gothenburg, and Malmö.

Using this approach, each SAMS was identified as either being an Iraqi enclave (that is, a neighborhood where the population of Iraqis was significantly greater than the surrounding areas) or not. We identified 15 Iraqi enclaves in Stockholm (average population of Iraqis in the enclaves: 10.2%), 9 in Malmö (average population of Iraqis in the enclaves: 15.5%), and 25 in Gothenburg (average population of Iraqis in the enclaves: 13.1%), for a total of 49 Iraqi immigrant enclaves (top panels, Figure 1). We identified only 2 outlier SAMS. As a comparison, Iraqi immigrants make up only 1.4% of the total sample population in these municipalities (Table 1).

**Table 1:** **Characteristics of SAMS** ^**a**^ **, population aged over 30 years in three Swedish municipalities, 2006–2010**

		Total immigrant population in quintiles				
	Overall	Q1	Q2	Q3	Q4	Q5
N SAMS	1,490	287	253	303	352	295
Population at risk	887,603	177,630	178,583	176,906	177,213	177,271
% Iraqi	1.4	0.1	0.2	0.2	0.8	5.7
% other immigrants	19.8	10.0	12.6	14.4	20.7	41.5
% Swedish	78.8	89.9	87.3	85.4	78.5	52.8
1st generation						
No	705,007	152,188	134,775	132,316	138,720	147,008
%	79.4	21.6	19.1	18.8	19.7	20.9
2nd generation						
No	182,596	25,442	43,808	44,590	38,493	30,263
%	20.6	13.9	24.0	24.4	21.1	16.6
Neighborhood deprivation index						
Mean (SD)	0.26 (2.02)	-1.55 (0.25)	-0.92 (0.14)	-0.41 (0.18)	0.61 (0.44)	3.61 (1.93)
Range	-2.55-10.14	-2 .55 - -1.16	-1.16 - -0.69	-0.69 - -0.04	-0.04 - 1.51	1.52 - 10.14
Total cases of incident diabetes mellitus	22,357	2,968	3,318	3,636	5,163	7,272

^a^SAMS: Small Area Market Statistics.

This approach to defining enclaves is consistent with, but more conservative than, Borjas’ [36] argument that an immigrant enclave is an area for which the probability that an immigrant resides in the neighborhood is greater than twice the probability of living in other areas. It also in line with conceptualizing residential segregation along multiple dimensions, specifically elements of evenness, exposure, clustering, and concentration, as argued by Massey and Denton in their study of “hypersegregation” of Blacks in US cities [37].

#### Diabetes status

Incident cases of DM from January 1, 2006 to December 31, 2010 were identified using the Swedish Prescribed Drug Register. This register includes all hospital, outpatient, and community (primary care) prescriptions in the nation beginning July 1, 2005. DM was indexed by a prescription for insulin or insulin analogs (Anatomical Therapeutic Chemical Classification System code A10A) or oral anti-diabetic agents (Anatomical Therapeutic Chemical Classification System codes A10B or A10X). In order to limit the outcome ascertainment to incident cases, prevalent cases of DM, indicated by prescription of these medications from July 1, 2005 to December 31, 2005, were excluded from the analysis.

#### Other covariates

Other individual-level covariates included age, sex, educational attainment (categorized as ≤9 years, 10–11 years or ≥12 years of schooling), income (categorized into quartiles), and 1st or 2nd generation status. We also accounted for neighborhood socioeconomic deprivation. As previously described [32, 38], a neighborhood deprivation index at the SAMS level was compiled from information on (a) percent with low educational attainment (defined as <10 years of schooling), (b) percent with low income (defined as less than 50% of median individual income), (c) percent unemployed, and (d) percent receiving social welfare assistance. These components were combined into a weighted sum, with higher values indicating more deprived neighborhoods. This index was categorized for analysis as low deprivation (>1 standard deviation (SD) below the mean), moderate deprivation (≥1 SD below and ≤1 SD above the mean) and high deprivation (>1 SD above the mean).

### Analysis

The goal of this analysis was to assess whether the cumulative incidence of DM in the Iraqi enclaves differed from that of the other SAMS in these municipal areas, and whether this effect was consistent across three subpopulations: 1st and 2nd generation Iraqi immigrants, 1st and 2nd generation immigrants from any other country, and native Swedes. Models were estimated separately for these three subpopulations. Cumulative incidence was calculated as the proportion of the population that developed DM over the 5-year follow-up period; individuals with prevalent DM at baseline were excluded from the analysis, so these cases all represent new-onset DM. Multi-level logistic regression was used to assess the association between residing in an Iraqi enclave and incidence of DM while accounting for the nesting of observations within SAMS. For each subpopulation, three models were estimated: Model 1: unadjusted, Model 2: adjusted for neighborhood deprivation, and Model 3: additionally adjusted for individual-level demographic and socioeconomic characteristics. Comparing the estimates from Models 1 and 2 illustrates the degree to which any effect of living in an Iraqi immigrant enclave has on DM independent from that of neighborhood deprivation. Comparing the estimates from Models 2 and 3 illustrates the degree to which individual-level risk factors contribute to DM risk, independent from those related to neighborhood context.

Random intercept multilevel logistic regression models were also used to estimate the intraclass correlation coefficient, the proportion of variance in the outcome attributable to differences between individuals in different SAMS (or classes) as opposed to differences between individuals within the same SAMS [39, 40]. The intraclass correlation coefficient ranges from 0 to 1; values close to 1 indicate that individuals within the same neighborhood are more highly correlated than individuals in different neighborhoods.

Analyses were completed using ArcGIS software (version 10), SAS (version 9.2) and MLwiN (version 2.021). All *P*-values refer to two-tailed tests.

## Results and discussion

Table 1 shows the sample characteristics overall and by SAMS that have been ordered according to the proportion of their population that is 1st or 2nd generation immigrants (not enclaves specifically). The table shows that as the proportion of Iraqi immigrants living in a SAMS increases, so does the proportion of other immigrant groups living in that SAMS; this is consistent with the notion that these areas can be broadly conceptualized as immigrant neighborhoods, not just Iraqi neighborhoods. The table also shows that as the immigrant population increases so does the socioeconomic deprivation of those neighborhoods. The bottom panels of Figure 1 and Table 2 show that the cumulative incidence of DM over the 5 year study period was twice as high in the Iraqi enclaves as compared to the other neighborhoods (4.7% vs. 2.3%). This elevated risk was seen in all subpopulations living in the enclaves, but the difference was least pronounced for the Iraqi immigrants.

**Table 2:** **Cumulative incidence of diabetes in enclaves vs. comparison SAMS** ^**a**^ **: 2006 - 2010**

Type of neighborhood	Number of SAMS	Neighborhood deprivation index	Cumulative incidence of diabetes (%)			
		Mean (SD)	Overall	Iraqi immigrants	Other immigrants	Native Swedes
Iraqi enclave	49	4.65 (1.54)	4.7	5.7	5.3	3.7
Non-enclave	1441	-0.09 (1.54)	2.3	5.2	3.4	2.1

^a^SAMS: Small Area Market Statistics.

Tables 3, 4 and 5 show the results of multi-level logistic regression models estimating odds of DM for the three subpopulations: Iraqi immigrants, other immigrants, and native Swedes. As shown by Table 3, despite the elevated risk of DM in the enclaves at an ecological level Iraqi immigrants living in an enclave have the same risk of DM as those living in other neighborhoods. Neighborhood deprivation and individual-level characteristics do not affect this estimate. In contrast, members of other immigrant groups living in an Iraqi enclave have elevated risk of DM (Table 4, Model 1), but this higher risk is entirely attributable to the high neighborhood deprivation of these areas (Model 2). Individual-level characteristics only modestly improve the explanatory power of the model. Finally, Table 5 shows that native Swedes living in Iraqi enclaves have significantly elevated risk of DM, and that this risk is substantially attenuated, but persists after accounting for neighborhood deprivation. Individual-level characteristics contribute more to the explanatory power of the model for this group relative to the two immigrant groups as illustrated by the increase in variance in DM explained by the model, but these individual-level variables did not attenuate the relationship between living in an enclave and odds of DM.

**Table 3:** **Relative odds of incident diabetes among Iraqi immigrants living in three Swedish municipalities: 2006 – 2010**

	Model 1		Model 2		Model 3	
	OR	95% CI	OR	95% CI	OR	95% CI
Iraqi living in an enclave *(ref. Iraqi not in an enclave)*	1.03	0.86, 1.24	0.99	0.79, 1.24	0.98	0.79, 1.23
Neighborhood deprivation *(ref. Low deprivation)*						
Level 2			0.82	0.61, 1.09	0.86	0.65, 1.14
Level 3			1.02	0.75, 1.38	1.06	0.78, 1.43
Level 4			1.20	0.89, 1.63	1.27	0.94, 1.71
Level 5 *(High deprivation)*			0.97	0.68, 1.38	0.99	0.69, 1.41
Age (years)					1.06	1.05, 1.07
Gender *(ref. Female)*					1.18	0.98, 1.41
Family income *(ref. High income)*						
Middle-high income					1.26	1.02, 1.57
Middle-low income					1.06	0.84, 1.34
Low income					1.04	0.80, 1.35
Education attainment *(ref. ≥ 12 years)*						
≤ 9 years					1.25	1.03, 1.51
10–11 years					1.16	0.90, 1.50
Generation status *(ref. First generation)*					0.31	0.08, 1.28
N	12,344		12,344		12,344	
Variance (SE)	0.070 (0.037)		0.064 (0.036)		0.045 (0.032)	
Explained variance (%)	1		10		37	
Intra class correlation	0.021		0.019		0.013

**Table 4:** **Relative odds of incident diabetes among other immigrants living in three Swedish municipalities: 2006 – 2010**

	Model 1		Model 2		Model 3	
	OR	95% CI	OR	95% CI	OR	95% CI
Other immigrant living in an enclave *(ref. Other immigrant not in an enclave)*	1.64	1.46, 1.84	1.05	0.95, 1.17	1.07	0.97, 1.19
Neighborhood deprivation *(ref. Low deprivation)*						
Level 2			1.12	1.01, 1.24	1.16	1.05, 1.28
Level 3			1.59	1.44, 1.75	1.59	1.45, 1.75
Level 4			1.88	1.70, 2.08	1.97	1.78, 2.17
Level 5 *(High deprivation)*			2.27	2.01, 2.57	2.42	2.14, 2.72
Age (years)					1.04	1.03, 1.04
Gender *(ref. Female)*					1.58	1.51,1.67
Family income *(ref. High income)*						
Middle-high income					1.08	1.00, 1.16
Middle-low income					1.15	1.07, 1.24
Low income					1.15	1.06, 1.24
Education attainment *(ref. ≥ 12 years)*						
≤ 9 years					1.00	0.93, 1.06
10–11 years					1.30	1.21,1.41
Generation status *(ref. First generation)*					0.54	0.48, 0.60
Variance (SE)	0117 (0.014)		0.037 (0.008)		0.027 (0.007)	
Explained variance (%)	24		76		82	
Intra class correlation	0.034		0.011		0.008

**Table 5:** **Relative odds of incident diabetes among native Swedes living in three Swedish municipalities: 2006 – 2010**

	Model 1		Model 2		Model 3	
	OR	95% CI	OR	95% CI	OR	95% CI
Native Swedish living in an enclave *(ref. Swedish not in an enclave)*	1.70	1.48, 1.95	1.25	1.11, 1.41	1.23	1.11, 1.36
Neighborhood deprivation *(ref. Low deprivation)*						
Level 2			1.12	1.03, 1.21	1.11	1.03, 1.19
Level 3			1.18	1.09, 1.28	1.19	1.11, 1.28
Level 4			1.58	1.46, 1.70	1.52	1.42, 1.62
Level 5 *(High deprivation)*			1.87	1.73, 2.01	1.79	1.67, 1.92
Age (years)					1.05	1.05, 1.05
Gender *(ref. Female)*					1.67	1.61, 1.73
Family income *(ref. High income)*						
Middle-high income					1.03	0.98. 1.08
Middle-low income					1.06	1.01, 1.12
Low income					1.05	1.00,1.10
Education attainment *(ref. ≥12 years)*						
≤ 9 years					0.56	0.53, 0.59
10–11 years					1.44	1.37, 1.52
Generation status *(ref. First generation)*					1.23	1.18, 1.28
Variance (SE)	0.132 (0.009)		0.069 (0.007)		0.037 (0.005)	
Explained variance (%)	10		53		75	
Intra class correlation	0.039		0.021		0.011	

## Conclusion

The primary finding from this study is that residing in an enclave is unrelated to risk of DM for either Iraqi immigrants or immigrants from other parts of the world, after accounting for potential confounders. In contrast, living in an Iraqi enclave is associated with elevated risk of DM among native Swedes, and this increased risk was attenuated but remained significantly elevated after accounting for neighborhood deprivation and individual-level socioeconomic and demographic characteristics. This is the largest prospective study to date to examine the relationship between immigrant enclaves and DM risk, and to our knowledge is the first to focus on Iraqi immigrants specifically.

These findings are broadly consistent with prior work on enclaves and DM and related conditions in that they show that the relationship between residential isolation/segregation is not uniform across all subpopulations. For example, living in a racially-segregated neighborhood is positively associated with obesity among US black women, but inversely associated with obesity for Mexican-American women [21]. More akin to our analysis, in a study of immigrants in Australia, Astell-Burt and colleagues [23] reported that living in a neighborhood with greater density of one’s own ethnicity was inversely associated with body mass index among migrants from the UK and Ireland but not any other immigrant group.

While we used a data-driven approach to empirically identify Iraqi enclaves, we feel confident that the neighborhoods identified here would also qualify as enclaves using a more sociological approach. As Logan et al. [13] articulated, immigrant enclaves are characterized both by the people that live in them (e.g., immigrants with limited material resources) and by their physical environment (e.g., lower resources). The percentage of Iraqis – as well as other immigrants – living in the enclave neighborhoods also strongly supports our categorization; the percentage of the population from any particular ethnic group in the US urban immigrant enclaves ranges from 8.1 to 69.8% [13], and our estimates are well within this range. However, we acknowledge that our reliance on quantitative data means that we have only a limited understanding as to how life differs for individuals (both immigrants and native Swedes) living in these areas as compared to predominantly Swedish settings. Our data did not provide clues to potential etiological pathways behind our findings and therefore we cannot draw causal inferences. However, the increased odds of incident DM among Swedes living in Iraqi enclaves remained after taking neighborhood deprivation into account, indicating that there is an adverse effect on DM of living in immigrant enclaves for native Swedes over and above the adverse effects of neighborhood deprivation.

These results should be interpreted in light of study limitations. It is possible that the relationship between neighborhood ethnic density and DM risk is not linear, and thus our findings using a categorical definition of enclaves – while consistent with sociological literature on immigrant neighborhoods – may not be directly comparable to reports using more continuous measures of population ethnic density. We also have no information on immigrants who repatriated to their country of origin. Our assessment of DM relies on pharmacy records and thus it is limited to only clinically-identified cases. While the vast majority (>85%) of DM cases are treated with some sort or medication [41], individuals managing their DM with lifestyle modification alone would not have been captured by this data source. If individuals who live in enclaves were less likely to access healthcare or use of pharmacologic treatment than those living in predominantly Swedish areas this would have the effect of biasing our results toward the null; however, we found that, prior to accounting for neighborhood deprivation, both other immigrants and native Swedes who lived in the enclaves had higher incidence of DM relative to other areas, and thus we do not feel this substantially impacted our inferences. This study also has a number of strengths. We were able to empirically identify Iraqi immigrant enclaves using GIS methods; while others have examined immigrant neighborhoods in Sweden [16], to our knowledge this is the first time this methodological approach has been used to identify these enclaves in this country. Second, our large sample size provided precision regarding the relationship between living in an immigrant enclave and DM risk. Finally, the prospective study design establishes the temporal relationship between neighborhood ethnic composition and onset of DM.

In conclusion, our findings indicate that ethnic composition is associated with the health of residents, but that this effect differs across ethnic group. Living in an enclave is not associated with increased odds of DM for immigrants, regardless of their nation of origin, despite the substantial socioeconomic deprivation of these neighborhoods. This suggests that living in an enclave may buffer the effects of neighborhood poverty for these groups. In contrast, even after accounting for neighborhood deprivation, living in an Iraqi enclave was associated with increased likelihood of DM for native Swedes. It is possible that the processes that influence place of residence differ for immigrants versus native Swedes (e.g., individual preferences, housing policies, limited affordable housing) which may explain these disparate relationships [12].

Finally, while cross-national comparisons must be made with caution, our results are broadly consistent with the work of LaVeist and others who have examined the health outcomes in racially-integrated neighborhoods in the US. For example, they report that whites living in racially-integrated urban neighborhoods have higher prevalence of DM than the national average for this group, and that there are negligible racial/ethnic differences in diabetes burden in these settings [42]. In this study we found that native Swedes living in Iraqi enclave had higher incidence of DM than those living in predominantly-Swedish areas, and that the difference in DM risk across migrant groups was smaller in the enclave neighborhoods than other areas. Future research should examine the psychosocial, economic, and political processes that may impact immigrant health, particularly for marginalized groups.
